# TESTIN Induces Rapid Death and Suppresses Proliferation in Childhood B Acute Lymphoblastic Leukaemia Cells

**DOI:** 10.1371/journal.pone.0151341

**Published:** 2016-03-17

**Authors:** Robert J. Weeks, Jackie L. Ludgate, Gwenn LeMée, Ian M. Morison

**Affiliations:** Department of Pathology, Dunedin School of Medicine, University of Otago, Dunedin, New Zealand; University of Sydney, AUSTRALIA

## Abstract

**Background:**

Childhood acute lymphoblastic leukaemia (ALL) is the most common malignancy in children. Despite high cure rates, side effects and late consequences of the intensive treatments are common. Unquestionably, the identification of new therapeutic targets will lead to safer, more effective treatments. We identified *TES* promoter methylation and transcriptional silencing as a very common molecular abnormality in childhood ALL, irrespective of molecular subtype. The aims of the present study were to demonstrate that *TES* promoter methylation is aberrant, to determine the effects of *TES* re-expression in ALL, and to determine if those effects are mediated via TP53 activity.

**Methods:**

Normal fetal and adult tissue DNA was isolated and *TES* promoter methylation determined by Sequenom MassARRAY. Quantitative RT-PCR and immunoblot were used to confirm re-expression of *TES* in ALL cell lines after 5’-aza-2’-deoxycytidine (decitabine) exposure or transfection with *TES* expression plasmids. The effects of *TES* re-expression on ALL cells were investigated using standard cell proliferation, cell death and cell cycle assays.

**Results:**

In this study, we confirm that the *TES* promoter is unmethylated in normal adult and fetal tissues. We report that decitabine treatment of ALL cell lines results in demethylation of the *TES* promoter and attendant expression of *TES* mRNA. Re-expression of TESTIN protein in ALL cells using expression plasmid transfection results in rapid cell death or cell cycle arrest independent of TP53 activity.

**Conclusions:**

These results suggest that *TES* is aberrantly methylated in ALL and that re-expression of TESTIN has anti-leukaemia effects which point to novel therapeutic opportunities for childhood ALL.

## Background

Childhood acute lymphoblastic leukaemia (ALL) is the most common malignancy of childhood and despite high cure rates [1] is a devastating disease for its young patients and their families. ALL treatment is intensive and side effects are common, with late consequences of the therapy of on-going concern [2]. Unquestionably safer, more effective and targeted treatments are desirable.

Childhood ALL has a peak incidence between 2 and 3 years [3] and is widely accepted to initiate prenatally, with retrospective studies of neonatal blood spots endorsing this prenatal origin [4]. ALL is characterised by the presence of clonal, immature lymphoblasts in the bone marrow. The mechanism by which these lymphoblasts have become abnormal is thought to involve multiple molecular events, including gene mutations and chromosomal translocations [5]. Many of the translocations result in expression of oncogenic proteins; for example, 15–25% of childhood ALL cases have a translocation between chromosomes 12 and 21, t(12,21)(p13;q22), resulting in expression of the oncogenic ETV6-RUNX1 fusion protein. Much work has focussed on these translocations and specific treatments targeted to translocations/fusion proteins have had remarkable success, e.g. Imatinib mesylate effectively targets the BCR-ABL1 fusion resulting from the t(9,22)(q34;q11) translocation [6]. However despite being important drivers for leukaemia, these translocations can be detected in blood from neonates who do not go on to develop ALL [7–9], indicating that these fusion proteins alone are insufficient to cause ALL.

Other potential leukaemogenic mechanisms have been investigated, such as epigenetic silencing. Many abnormally methylated genes have been reported in ALL, for example, *CDKN2B* promoter methylation has been repeatedly described in ALL leading to claims that this methylation is involved in leukaemogenesis [10]. However, promoter methylation can occur as a non-specific “bystander” event affecting genes that are already silent. Keshet *et al*. reported that of 106 genes whose promoters were methylated in colon cancer cell lines, 91 were already inactive in normal colon [11]. Similarly, Sproul *et al*. documented 120 genes that were significantly methylated and repressed in primary breast cancers and cell lines, of which greater than 90% were not normally expressed in the cell of origin and reflected the epigenetic state of the cell of origin and were unrelated to tumourigenesis [12].

Methylation of the *TES* promoter is a common feature of ALL. We reported that dense, biallelic methylation of the *TES* promoter and subsequent silencing of expression was a common molecular abnormality in childhood ALL, occurring in over 90% of B ALL (n = 100) and over 70% of T ALL (n = 27) cases, irrespective of ALL sub-type classification [13]. Furthermore our results have been confirmed in subsequent independent studies; for example, *TES* expression was downregulated in B-lineage ALL [14, 15]; and *TES* promoter methylation was demonstrated to be specific to ALL in a methylation microarray study of haematological neoplasms [16].

*TES* promoter methylation and transcriptional silencing has also been reported in glioblastoma [17–19], breast [20, 21], endometrial [22], gastric [23], uterine [20], head and neck [24], ovarian [25] and prostate [26] tumours.

*TES* is a putative tumour suppressor gene located on chromosome 7q31 that encodes the highly conserved TESTIN protein. TESTIN is a 421 amino acid protein, containing a PET domain and three LIM domains. LIM domains are protein-binding, zinc fingers and are frequently observed in proteins involved in forming multi-protein complexes [27]. LIM-domain containing proteins have important roles in many cellular processes, including development [28, 29] and cancer [30].

TESTIN function is likely to be mediated via its protein interactions. Protein partners for TESTIN have been identified and include, for example, members of the focal adhesion complex, such as Zyxin, ENA/VASP and paxillin [31–34]. Over-expression of TESTIN results in increased cell adhesion and decreased migration [35]. Immunohistochemistry confirmed localization of TESTIN to the focal adhesion complex and possible localization to the nucleus [36]. Although, interactions of TESTIN with nuclear proteins have not been reported, many LIM domain proteins are known to shuttle between the cytoplasm and nucleus; for example, Zyxin [37], FHL2 [18] and others [27].

Recent studies have investigated re-expression of TESTIN in *TES*-negative cancer cell lines, such as breast, uterine [20], ovarian [25] and endometrial cancer [22]. These reports consistently demonstrate the tumour suppressive effects of TESTIN re-expression, such as reduced proliferation and apoptosis in *TES*-negative cell lines and tumours.

In this study we confirm that the *TES* promoter is not methylated in normal adult and fetal tissues. We demonstrate that decitabine treatment of ALL cell lines results in *TES* promoter demethylation and re-expression. In addition, we show that *TES* expression after plasmid transfection results in decreased proliferation and increased cell death in ALL cell lines, similar to results obtained with other cancer cell lines. Finally, we show that TESTIN induces these effects rapidly after re-expression and that these effects are independent of TP53 protein induction.

## Material and Methods

### Sequenom MassARRAY EpiTYPER Analysis

Human fetal tissue was obtained from terminations of pregnancy under the provisions of an Otago Ethics Committee (New Zealand) approval. Dr I. Morison, K. Drake, Prof A. Reeve–“Characterisation of early embryonic fetal lymphoid development”. Otago Ethics Committee, 18 August 2003 [03/05/033]. (Originally, we obtained Ethics approval from the Otago Ethics Committee (New Zealand). Regional approval for this application was also obtained from the Wellington Ethics Committee. Approval was given to collect the products of conception from 5–12 week terminations of pregnancy at Dunedin and Wellington Area Hospitals and no identifying information was associated or sent with these samples.

The human adult somatic tissues (colon, kidney, heart, liver, lung, lymph node, muscle and thyroid) and tumour samples (breast cancer, colon cancer, Wilms tumour and ALL) were obtained at autopsy or from healthy volunteers (peripheral blood), following informed written consent from next of kin.

The cell lines used for this study were purchased from the American Tissue Type Collection (CCRF-CEM, HL60, Jurkat, K562, MOLT4 and Raji) by the Cancer Genetics Laboratory (University of Otago) or from the RIKEN BioResource Center, Japan (NALM6).

Normal fetal and adult tissue samples, tumours and cell lines were analysed for *TES* promoter methylation by Sequenom MassARRAY, as before [13]. Data were analysed using MassARRAY EpiTYPER software. Sequenom analysis of the *TES* promoter region resulted in 19 identified fragments, spanning 48 CpG sites. Mean methylation values were calculated for the amplified region.

### RT-PCR

Qualitative and quantitative RT-PCR assays were performed as previously described [13]. To ensure the absence of contaminating genomic or expression plasmid DNA in the samples, reactions without reverse transcriptase were always performed in parallel.

### Expression Plasmid Preparation

Full length *TES* coding sequence was amplified from PBL cDNA, using EXPAND High-Fidelity PCR System (Roche) and primers specific for the translational start and stop sites (Forward primer: 5’-GGG CAT AAG CTT CCA TGG ACC TGG AAA ACA AAG TGA AC-3’ and Reverse primer: 5’-GGG CAT GGA TCC GAT AGC TAT GGC TCG ATA CTT CTG-3’). *TES* cDNA was cloned into pcDNA3.1 or pEGFP-C1 (in-frame and downstream of EGFP coding sequence). We confirmed that the *TES*-expression plasmid sequences were correct and in-frame. Multiple plasmid preparations were prepared using endotoxin-free maxi preparation kits and combined.

### Cell culture

Lymphoblastoid cell lines were cultured as recommended by the ATCC or the RIKEN BioResource Center. 5’-aza-2’-deoxycytidine (decitabine; Sigma-Aldrich) was freshly prepared and added directly to cells each day. WST-1 (Roche) assay was used to determine cell number and viability during decitabine treatment and cells were collected daily for DNA and RNA isolation.

Transfection of haematopoietic cells was achieved using the NEON Transfection system (Life Technologies Ltd). We routinely achieved 30–70% transfection efficiency with this system as determined using the control GFP-reporter plasmid (pEGFP-C1).

### Flow cytometry

All flow cytometry was performed with a Gallios™ flow cytometer (Becton-Dickinson Ltd). All stains were used as per manufacturer’s instructions. Control experiments were performed in order to calculate appropriate spectral compensations. Cell sorting for GFP-positive, transfected cells was performed using a BD FACSAria flow sorter.

Flow cytometry assays were as follows: (i) Transfection efficiencies were calculated using GFP fluorescence; (ii) Cell death or cell viability was monitored using Violet Live/Dead (VLD; Life Technologies Ltd) staining; (iii) Apoptosis was measured with annexin V labelling and VLD staining; (iv) Cell proliferation was monitored after staining of cells with Violet Proliferation Dye (VPD; Life Technologies Ltd) (no toxicity due to VPD-staining of the cell lines was observed) (v) Cell cycle analysis was performed on GFP-sorted and ethanol-fixed cells after RNAse-digestion and 7-AAD staining, using the Watson Pragmatic model (FlowJo software v8.8.7).

Flow cytometry data were analysed using FlowJo (TreeStar LLC, USA) or Kaluza (Becton Dickinson) software.

### Immunoblot

Protein lysates were prepared from cell pellets resuspended in RIPA buffer and stored at -80°C. Protein (40 μg) was electrophoresed and blotted to nitrocellulose using the iBlot system (Life Technologies). Membranes were blocked with 5% non-fat milk/PBS-T before incubation with antibodies specific for TESTIN (1:1000, rabbit polyclonal, Abcam), TP53 (1:1000, mouse monoclonal, 1C12, Cell signalling), CDKN1A (1:500, mouse monoclonal, sc-6246, Santa Cruz Biotechnology) or α-actin (1:15,000, a1978, Sigma-Aldrich).

## Results

### *TES* promoter methylation in normal tissues

Previously, we reported dense, biallelic promoter methylation and transcriptional silencing of *TES* in childhood ALL samples (>90% of B and >70% of T ALL), with matched remission bone marrow and normal peripheral blood leukocytes (PBL) being unmethylated and transcriptionally active [13]. *TES* transcriptional silencing by promoter methylation has been documented in many tumour types, including breast, uterine and glioblastoma cancers [17–19, 20, 21,].

**Fig 1:**
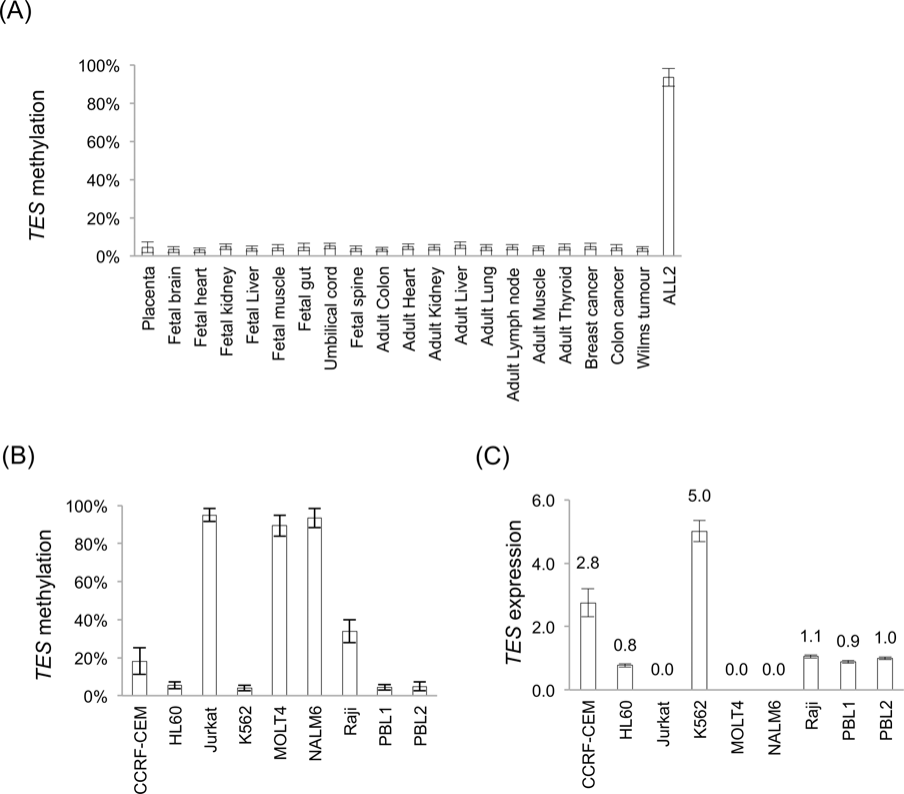
*TES* promoter methylation and expression. Sequenom MassARRAY methylation data of (A) normal, somatic tissues, and (B) lymphoblastoid cell lines (95% confidence intervals are shown). (C) *TES* expression levels by quantitative RT-PCR were calculated relative to *B2M* expression and normalised to relative expression levels in the normal peripheral blood leukocyte sample (PBL2) (error bars are standard deviations).

Using Sequenom MassARRAY, the *TES* promoter was found to be unmethylated in all normal fetal and adult tissues tested (Fig 1A and S1 File). As previously reported, the *TES* promoter was densely methylated in B ALL [13] and in a B ALL (NALM6) cell line. We did not observe *TES* methylation in the single examples of breast, colon and Wilms tumours tested.

### *TES* promoter methylation and expression in lymphoblastoid cell lines

Previously, we documented *TES* promoter methylation and transcriptional silencing in several lymphoblastoid cell lines [13]. In this report, we investigated methylation of the *TES* promoter and *TES* expression in additional lymphoid-derived cell lines.

We observed dense methylation of the *TES* promoter in Jurkat (T ALL), MOLT4 (T ALL) and NALM6 (B ALL), but only partial methylation in the CCRF-CEM (T ALL) cell line (Fig 1B). Previously, Tatarelli *et al*. reported that CCRF-CEM cells have a mutation in the *TES* coding sequence (R266H)[38] and we were able to confirm the presence of this mutation (data not shown). The effect of this mutation on the activity or function of TESTIN is unknown. We observed no *TES* coding mutations in the other cell lines or in primary or xenograft ALL samples (data not shown) and we are unaware of any *TES* coding sequence mutations having been reported in B or T ALL cases.

Both HL-60 (acute promyelocytic leukaemia) and K562 (chronic myelogenous leukaemia) cell lines had unmethylated *TES* promoters and no coding mutations. Previously, using CoBRA (*Co*mbined *B*isulfite *R*estriction *A*ssay), we had determined that the Raji (Burkitt lymphoma) cell line was hemi-methylated at the *TES* promoter [13], and this was confirmed with the results from Sequenom MassARRAY.

Using qRT-PCR, we quantified *TES* expression in the cell lines and PBL samples (Fig 1C). In summary, cell lines with densely methylated *TES* promoters do not express *TES* RNA (Jurkat, MOLT4 and NALM6); whereas cell lines and normal PBL samples express *TES* RNA and are largely unmethylated at the *TES* promoter.

These results confirm our previous observation of the inverse relationship between promoter methylation and expression for the *TES* gene [13].

### 5’-Aza-2’-deoxycytidine treatment of lymphoblastoid cell lines

To investigate the effect of promoter demethylation on *TES* expression, we treated NALM6, MOLT4 and Jurkat (methylated) cell lines with 5’-aza-2’-deoxycytidine (decitabine), a cytidine analogue that induces global DNA demethylation via inhibition of DNA methyltransferase activity [39].

**Fig 2:**
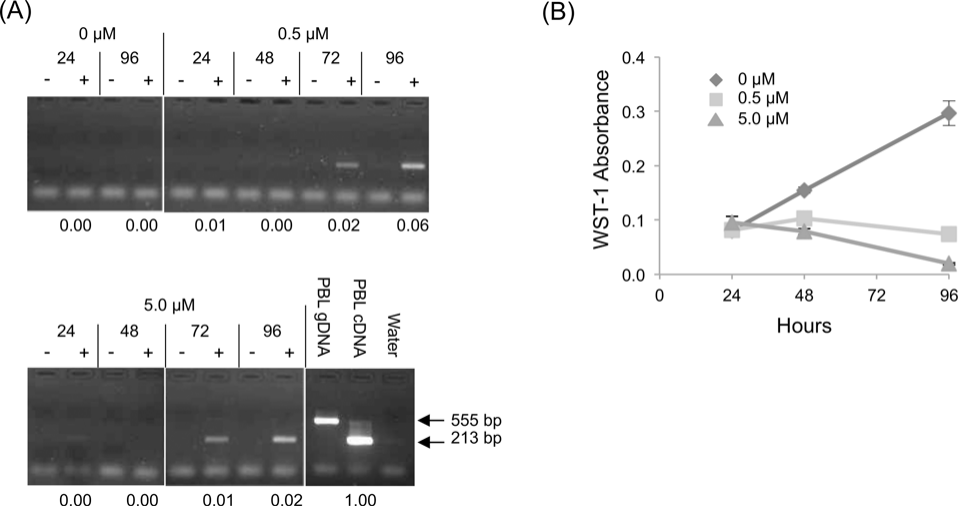
*TES* promoter demethylation and decreased NALM6 cell viability after decitabine exposure. NALM6 cells were exposed to decitabine (0, 0.5 and 5.0 μM) for 24, 48, 72 and 96 hours. (A) *TES* qualitative RT-PCR: total RNA was mock (-) or reverse transcribed (+) before *TES*-specific amplification with exon-specific primers (exons 5 and 6). Expected PCR product sizes for cDNA and genomic DNA were 213 bp and 555 bp, respectively. *TES* expression levels, as measured by quantitative RT-PCR assay and calculated relative to PBL cDNA, are recorded under each lane. (B) Viable NALM6 cell number was measured by WST-1 assay (error bars are standard deviations).

Cells treated with decitabine were counted and collected for RNA and DNA every 24 hours. We observed demethylation of the *TES* promoter and re-expression of *TES* RNA after decitabine exposure confirming that aberrant *TES* promoter methylation is the mechanism by which *TES* expression is silenced in ALL (Fig 2A and S1A Fig). In addition, decitabine exposure is known to be cytotoxic and we demonstrate that cells exposed to increasing amounts of decitabine fail to proliferate (Fig 2B and S1B Fig).

### TESTIN expression in lymphoblastoid cells

To better investigate the effects of *TES* expression in lymphoblastoid cells, full length *TES* cDNA was amplified and cloned into mammalian expression plasmids (see Materials and Methods). Re-expression of *TES* was achieved using transient plasmid transfections and the NEON electroporation system, with which we achieved efficient transfections (30–70% efficiency).

Initially, we performed experiments with both *TES* (pcDNA3.1-based) and EGFP-*TES* fusion (pEGFP-C1 based) expression plasmids, but as similar results were observed with both plasmids (S2 Fig), we proceeded exclusively with the pEGFP-TES plasmid, which permitted visualisation of transfected cells (GFP positive) by fluorescence microscopy and flow cytometry.

Cells were transfected with either PBS (mock), pEGFP-C1 (control EGFP-expression plasmid) or pEGFP-TES expression plasmid.

**Fig 3:**
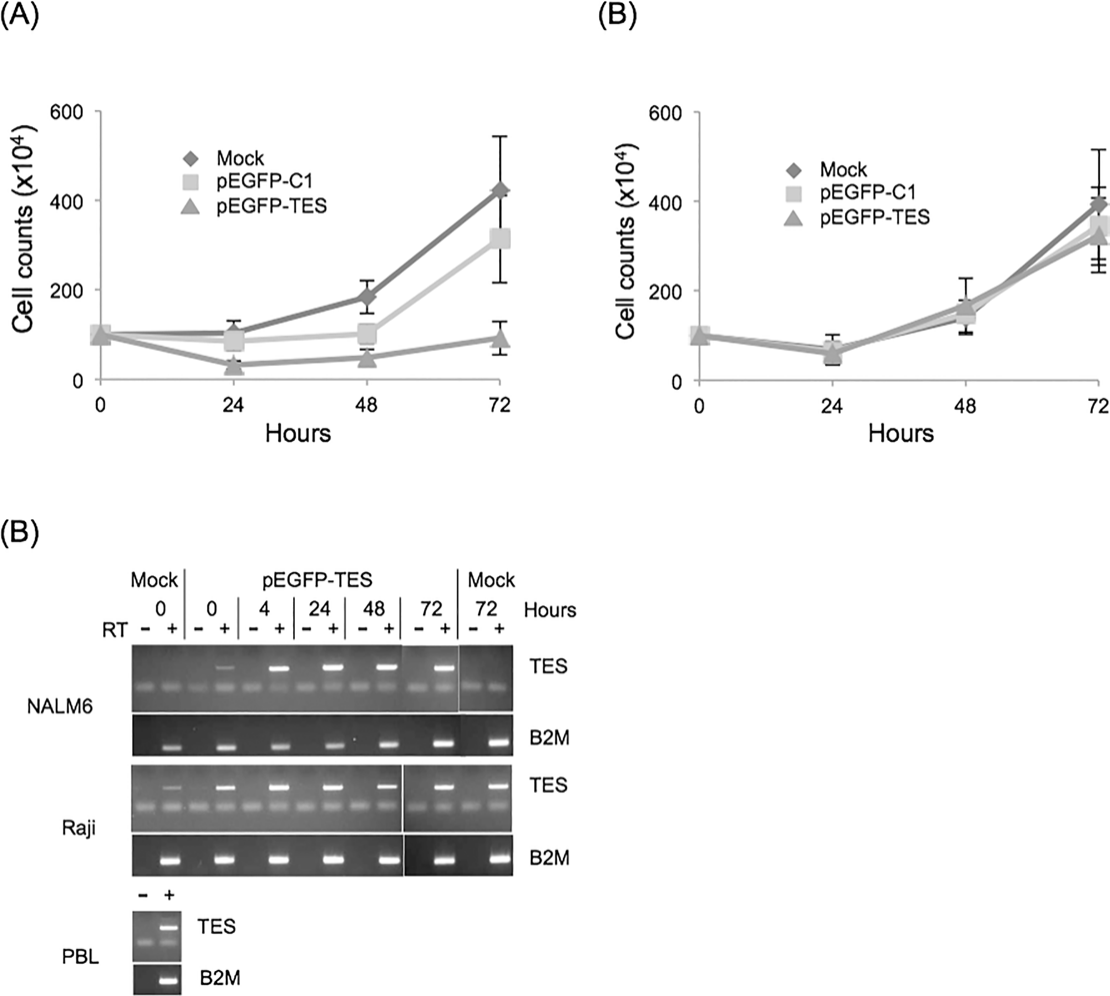
*TES* transfection induces rapid expression and reduced proliferation in NALM6 cells compared to Raji cells. Cells were transfected with mock, pEGFP-C1 or pEGFP-TES expression plasmids, cultured and counted after trypan blue staining; (A) NALM6 (n = 5) and (B) Raji (n = 3) cells (error bars are standard errors). (C) RT-PCR of transfected cells showing rapid, robust *TES* expression after pEGFP-TES transfection. PBL cDNA and genomic DNA were included as controls. In addition, RT negative samples were included to confirm removal of contaminating transfected plasmid and genomic DNA.

Trypan blue stained cell counts demonstrated that there were fewer surviving NALM6 cells after pEGFP-TES transfection and that those cells failed to proliferate compared to mock- or pEGFP-C1 control-transfected cells (Fig 3A). Similar results were obtained after transfection with other *TES*-negative cell lines (Jurkat and MOLT4)(S3 Fig). In contrast, mock, control or pEGFP-TES transfected Raji cells were viable and proliferated at similar rates, indicating that exogenous *TES* expression did not induce cell death or reduce proliferation of Raji cells (Fig 3B). As we were focussed on *TES* re-expression in B cells, we proceeded with transfections of NALM6 (B ALL; *TES*-negative) and Raji (mature B cell lymphoma; *TES*-positive) cell lines.

Using RT-PCR, we confirmed *TES* RNA transcript expression in Raji and NALM6 transfections (Fig 3C). As expected, we detected *TES* transcript in both mock- and pEGFP-TES-transfected Raji cells, confirming that Raji cells express *TES* RNA. We confirmed that NALM6 cells do not express endogenous *TES* RNA (“Mock” lanes, Fig 3C), but that *TES* transcript could be detected within 15 minutes of transfection with pEGFP-TES expression plasmid (Time 0, Fig 3C).

### Increased cell death after pEGFP-TES transfection

The failure of pEGFP-TES transfected NALM6 cells to proliferate, as demonstrated by the trypan blue viable cell counts (Fig 3A), can result from increased cell death, a reduction in proliferation or both.

**Fig 4:**
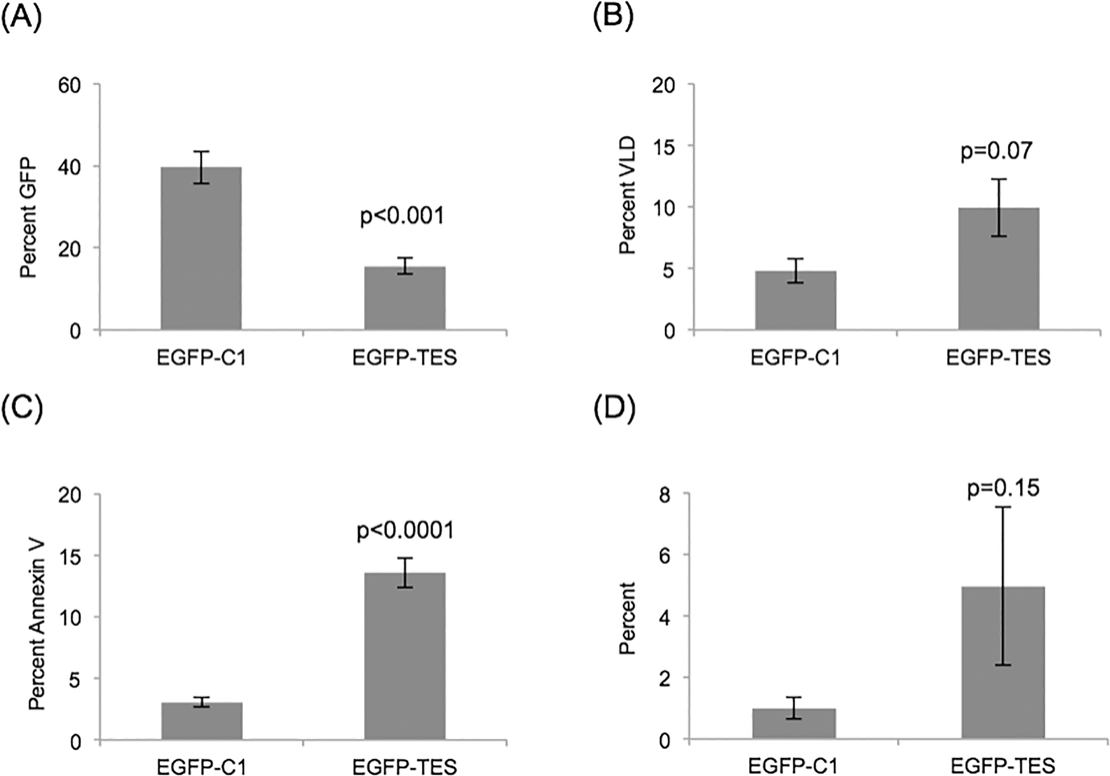
Flow cytometry results showing reduced numbers of GFP-positive NALM6 cells and increased cell death after pEGFP-TES transfection. Transfected NALM6 cells (24 hours post-transfection) were labelled with Annexin V and VLD and analysed by flow cytometry. (A) Decreased numbers of GFP-positive NALM6 cells were present after pEGFP-TES transfection (p<0.001). After gating for GFP fluorescence, increased numbers of cells are (B) VLD positive (p = 0.07), (C) Annexin V positive (p<0.0001) and (D) Annexin V positive and VLD negative (p = 0.15) (n = 5; error bars are standard errors; significance values were calculated using unpaired, two-tailed, Student’s t-Test).

Fewer GFP-positive NALM6 cells were present after pEGFP-TES transfection than with pEGFP-C1 control transfection and this reduction in GFP-positive cells after pEGFP-TES transfection did not occur with Raji cells (Fig 4A and S4 Fig). To investigate cell death, we stained transfected cells (24 hours post-transfection) with viability stain (Violet Live/Dead (VLD); Life Technologies) and measured the proportion of dead cells using flow cytometry. More NALM6 cells were dead (VLD positive) after pEGFP-TES transfection than with pEGFP-C1 control or mock-transfections (Fig 4B and data not shown) confirming that EGFP-TES expression results in increased cell death.

Next, we investigated whether the observed cell death was due to apoptosis. Transfected cells (24 hours post-transfection) were labelled with Annexin V and VLD and analysed by flow cytometry. Annexin V binds to phosphatidylserine (PS) exposed on cellular membranes during cell death and cells undergoing apoptosis expose PS before losing the membrane integrity characteristic of cell death, i.e. would appear as annexin v positive and VLD negative. We observed increased numbers of annexin V positive cells (Fig 4C; p<0.0001) and an increase in “early apoptotic cells” (annexin V positive and VLD negative) between EGFP-TES and control transfected NALM6 cells was observed in individual experiments (data not shown); however statistical significance was not achieved (p = 0.15) after averaging experimental results (n = 5) (Fig 4D). Our results confirm previously published results demonstrating increased cell death from *TES* re-expression experiments in *TES*-silenced cancer cell lines [20, 22, 25].

### Anti-proliferative activity

From the flow cytometry and trypan blue cell count results, it was clear that most pEGFP-TES transfected NALM6 cells were dead or dying within 24 hours (Fig 4), but that those transfected cells that escaped cell death were not proliferating (Fig 3A).

To monitor cell proliferation, we stained cells with Violet Proliferation Dye (VPD; Life Technologies) and measured VPD fluorescence intensities using flow cytometry. NALM6 cells labelled with VPD were transfected and cultured as before. VPD fluorescence of transfected cells was measured at 24, 48 and 120 hours. Untransfected NALM6 cells proliferate rapidly, with VPD median fluorescence intensity (MFI) halving every 24 hours (data not shown).

**Fig 5:**
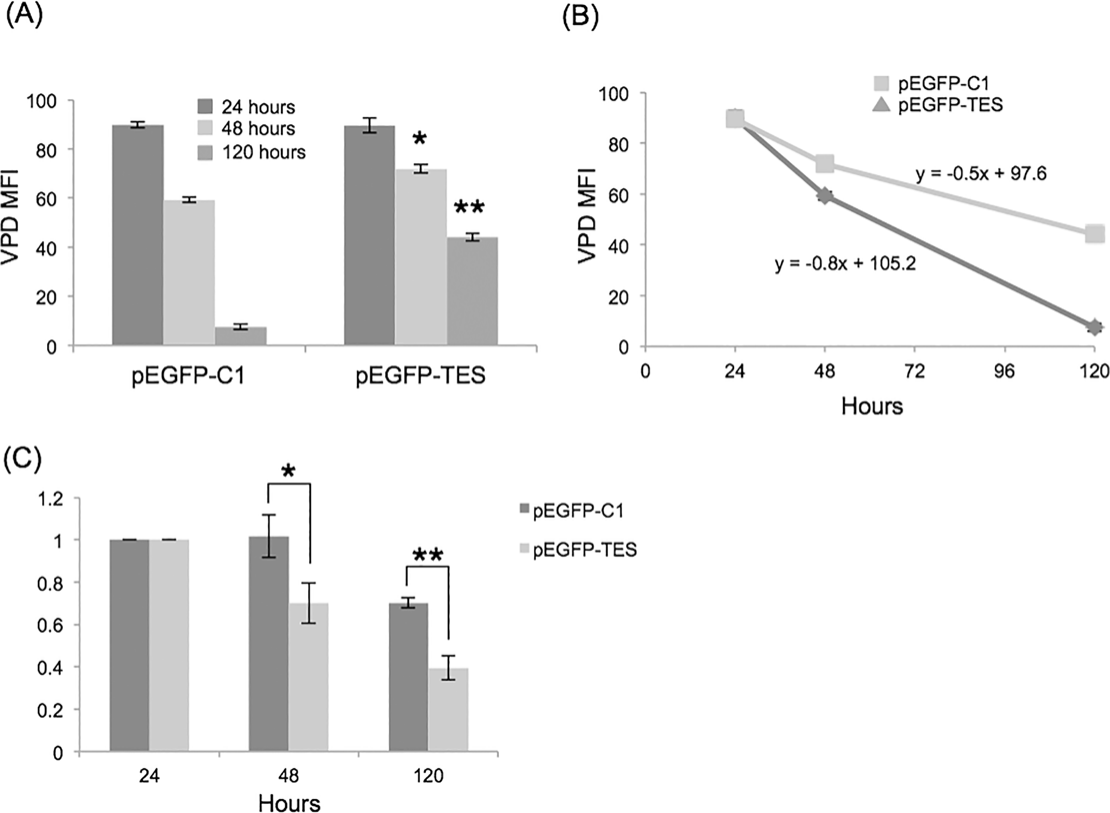
Reduced proliferation of pEGFP-TES transfected NALM6 cells. (A) Average VPD MFI for GFP-positive, transfected cells at 24, 48 and 120 hours were compared (n = 3). pEGFP-TES transfected NALM6 cells have higher average VPD MFI than GFP-negative (data not shown) or control transfected NALM6 cells, indicating that these cells have undergone fewer cell divisions (unpaired, two-tailed Student’s T-test; * p = 0.003, ** p<0.0001). (B) Proliferation rates of GFP-positive, transfected cells clearly showing reduced proliferation of pEGFP-TES transfected compared to control-transfected cells (line equations were calculated using MS Excel). (C) Reduction in GFP-positive cells over time, calculated relative to 24 hours. Significance was determined between control- and EGFP-TES-transfected cell proportions at 48 (* p = 0.07) and 120 hours (** p = 0.009)(unpaired, two-tailed Student’s T-test).

Differences in VPD MFI were apparent between control and pEGFP-TES transfected cells at 48 hours (p = 0.003) and 120 hours (p<0.0001), indicating that pEGFP-TES transfected cells had completed fewer cell divisions than control transfected or untransfected cells (Fig 5A and data not shown). Assuming that VPD MFI will halve after each cell division, we calculated that pEGFP-TES transfected cells had divided 1–2 times, compared to 3–4 times for control transfected and 4–5 times for untransfected cells over 120 hours. Comparison of VPD MFI plotted versus time for each transfection demonstrates that pEGFP-TES transfected cells proliferate significantly slower than untransfected or control-transfected cells (Fig 5B).

Despite transfected cell numbers being low, we monitored loss of pEGFP-TES cells by comparing the proportion of GFP-positive cells relative to cell numbers at 24 hours (Fig 5C). At 48 hours, the proportion of pEGFP-TES transfected cells was reduced by 30% and even fewer pEGFP-TES transfected cells were present at 120 hours, as the transfected cell population die or proliferate slower than untransfected cells. For control-transfected cells, the proportion of transfected to untransfected cells was the same after 48 hours confirming that control-transfected cells proliferate at similar rates to untransfected cells. Transiently transfected cells are expected to lose expression over time and we do observe a decrease in the proportion of GFP-positive control cells at 120 hours post-transfection, likely due to this loss of expression. Overall, comparing GFP-positive cell numbers between control- and EGFP-TES-transfections confirmed that EGFP-TES transfected cells proliferate slower than control or untransfected cells.

### Cell cycle arrest

**Fig 6:**
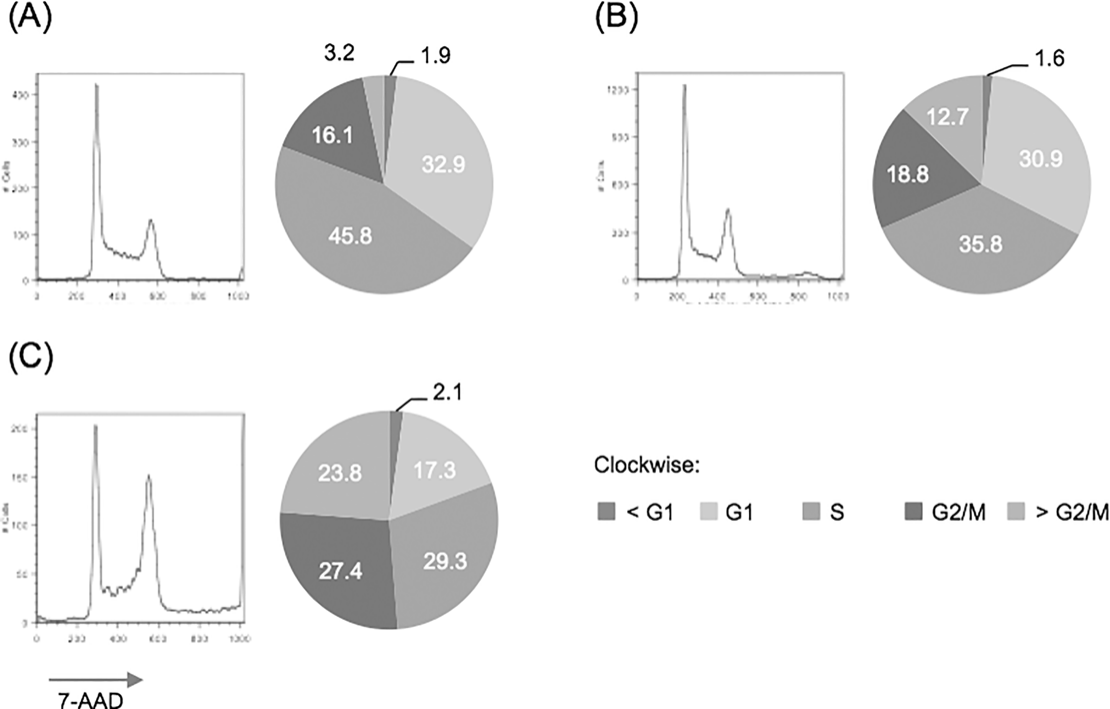
Cell cycle analysis of GFP-sorted, transfected cells. GFP-positive NALM6 cells at 48 hours post-transfection were fixed with ethanol before 7-AAD staining. DNA content (7-AAD intensity) was measured using a flow cytometer; (A) untransfected, (B) pEGFP-C1, or (C) pEGFP-TES, (*left*: cell cycle profile histograms) and analysed using the Watson Pragmatic Cell Cycle module (FlowJo) and proportion of cells in each cell cycle phase calculated (*right*: pie charts).

We investigated the anti-proliferative effect of TESTIN expression on the kinetics of the cell cycle by measuring DNA content after 7-AAD staining (Fig 6). NALM6 cells were transfected and cultured for 24 hours, before sorting GFP-positive cells with a FACSAria cell sorter. Forty-eight hours post-transfection, GFP-sorted cells were fixed with cold 70% ethanol and stained with 7-AAD after RNAse digestion. DNA content of cells was measured by flow cytometry and cell cycle profiles were modelled using FlowJo software (TreeStar).

NALM6 cells divide rapidly (doubling times are <24 hours) and untransfected cells have “active” cell cycle profiles with approximately 60% of cells replicating their DNA (S-phase) or preparing to divide (G2-phase) (Fig 6A). Overall control-transfected cells had a DNA-content profile similar to that of untransfected cells (comparing G1 and G2/M peak heights), indicating that their cell cycle profile was largely unaffected by pEGFP-C1 transfection (Fig 6B). However, we did observe fewer cells in S-phase (35.8% compared to 45.8% of untransfected) and some cells with abnormal DNA content (12.7% compared to 3.2% had DNA content >4N), presumably due to the effects of electroporation. Conversely, transfection with pEGFP-TES resulted in profound changes to the measured DNA profile, particularly prominent being the larger G2/M peak (Fig 6C), More specifically, fewer cells are observed in the G1- (17.3% compared to 30.9% control-transfected) and S-phases (29.3% compared to 35.8% control-transfected) and more cells are in G2/M (27.4% compared to 18.8%). Overall more than 50% of pEGFP-TES transfected cells have DNA content greater or equal to 4N, which is suggestive of a cell cycle block in G2/M. And the presence of a large population of pEGFP-TES transfected cells with DNA content greater than 4N (23.8% compared to 12.7% of control-transfected) suggests that that these cells were unable to undergo cell division. Using confocal microscopy we were able to document the presence of bi-nucleated, pEGFP-TES transfected NALM6 cells (see S5 Fig), thus confirming that some transfected cells are unable to complete cytokinesis after completing telophase. We did not observe sub-G1 pEGFP-TES transfected cells, demonstrating that these cells were not undergoing apoptosis at this time (48 hours post-transfection).

### TP53 protein pathway

As TESTIN expression induces cell death and cell cycle arrest, we investigated the effect of TESTIN expression on the TP53 pathway. TP53 can induce cell cycle arrest, in part due to its ability to induce the expression of CDKN1A [40]. Several potential interactions between TP53 and TESTIN, via known TESTIN binding partners, have been reported [37, 41]. Importantly, ELL2 is able to bind TP53 and inhibit the transactivation activity of TP53 [41] and thus we propose that TESTIN may have a role in controlling TP53’s ability to transactivate *CDKN1A*.

**Fig 7:**
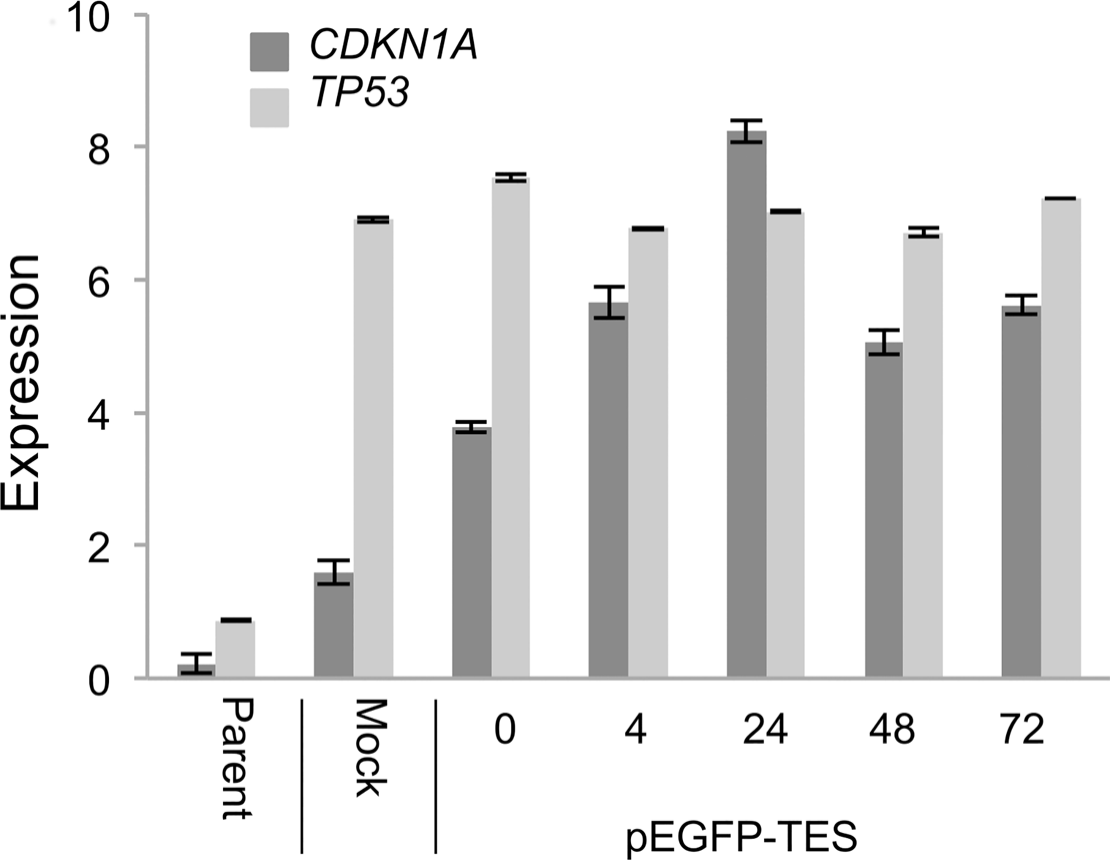
*CDKN1A* and *TP53* expression levels in pEGFP-TES transfected NALM6 cells. *TP53* and *CDKN1A* transcript levels were measured after transfection by quantitative RT-PCR. Expression levels were normalised to *B2M* expression (error bars are standard deviations).

We quantified *CDKN1A* transcript in transfected cells using qRT-PCR. We observed a rapid increase in *CDKN1A* transcript following electroporation with pEGFP-TES and a smaller induction after mock electroporation (Fig 7).

In addition, we demonstrated that *TP53* transcript levels were raised in all transfected samples, presumably due to induction after electroporation (Fig 7).

**Fig 8:**
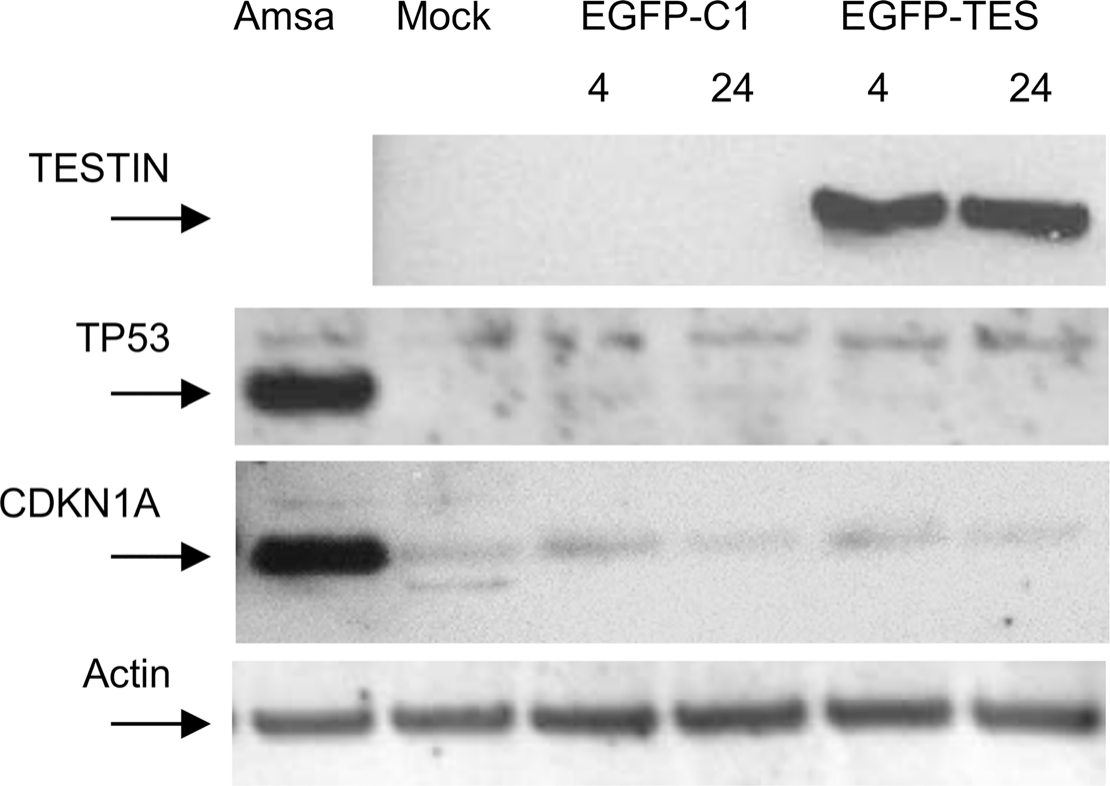
Immunoblots of transfected and amsacrine-treated NALM6 cells. Forty μg of protein lysate from transfected and amsacrine-treated NALM6 cells were immunoblotted. EGFP-TESTIN fusion protein was detected using rabbit anti-TESTIN polyclonal antibody (Abcam: ab78499), and results showed rapid expression after transfection. Anti-TP53 monoclonal antibody (Cell Signalling) and anti-CDKN1A antibody (Santa Cruz Biotechnology) demonstrate lack of TP53 or CDKN1A induction after transfection, but robust induction after amsacrine exposure. Anti-α actin antibody was used to confirm equal protein loading.

Immunoblotting of protein lysates from transfected cells demonstrated rapid and robust expression of EGFP-TES fusion protein at 4 hours post-transfection (Fig 8). We observed no increase in CDKN1A protein levels in pEGFP-TES transfected cells, compared to the control transfected NALM6 cells (Fig 8). However, we did observe a non-specific increase in CDKN1A protein levels at 4 hours post-transfection, presumably due to the cellular response induced after DNA electroporation, although this response is independent of an increase in TP53 protein. NALM6 cells are reported to possess wild-type TP53 and we confirm that they have a normal *TP53* response to DNA damage, with robust induction of TP53 and CDKN1A after exposure to Amsacrine (Topoisomerase II inhibitor) [42]. In summary, neither TP53 nor CDKN1A proteins were induced after pEGFP-TES transfection (Fig 8).

## Discussion

*TES* is located on the long arm of chromosome 7, in a region frequently displaying loss of heterozygosity in many tumours [26, 43, 44], including myelodysplastic syndrome and myeloid leukaemias [45–48]. *TES* as a putative tumour suppressor gene is likely to be transcriptionally active in normal tissues. Multiple reports document transcriptional silencing of the *TES* gene in many tumour types and that this silencing is accompanied by promoter methylation [17–26]. Using Sequenom MassARRAY, we show that the *TES* promoter is unmethylated in all normal fetal and adult tissues tested (Fig 1A and S1 File), supporting the proposition that *TES* is transcriptionally active and constitutively expressed in normal tissues and that the frequently observed *TES* promoter methylation present in many tumours is aberrant.

Previously, we reported dense, biallelic and clonal methylation of the *TES* promoter in childhood ALL cases, irrespective of subtype classification and that *TES* promoter methylation resulted in *TES* transcriptional silencing in ALL xenografts and cell lines [13]. In this study, we show that the *TES* promoter is densely methylated in additional T and B ALL cell lines (Fig 1B; Jurkat, MOLT4 and NALM6) and confirmed methylation in ALL. We demonstrate that these *TES*-methylated, ALL cell lines do not express *TES* RNA, confirming that promoter methylation silences RNA transcription at the *TES* promoter (Fig 1C). We also show that the non-ALL lymphoblastoid cell-lines tested were unmethylated at the *TES* promoter and expressed *TES* RNA transcript similar to normal PBL expression levels. We confirmed that CCRF-CEM, the only ALL cell line tested that expresses *TES* RNA, does possess a *TES*-coding mutation (data not shown), the consequence of which is unknown [38].

Sproul *et al*. observed that most genes aberrantly methylated in tumours are not expressed in normal matched tissues and that demethylation of these genes does not result in expression in tumours. Thus, they conclude that many gene promoters methylated in tumours reflect the repressed expression status of the tissue of origin and are unrelated to tumourigenesis [12]. Using decitabine treatment, we demonstrate rapid removal of methylation with concomitant expression of *TES* transcript showing that *TES* can be expressed in ALL cells and supporting the hypothesis that *TES* is normally expressed in the ALL cell-of-origin and that methylation of the *TES* promoter contributes to leukaemogenesis. Two large-scale studies support this hypothesis. Firstly, analysis of methylation microarray data of 387 haematopoietic neoplasms confirms that *TES* promoter methylation is frequently observed in B and T ALL samples (n = 37/42 and 14/18, respectively) and less frequently observed in other haematopoietic tumours, such as MDS (n = 4/13) and AML (n = 25/116), but is not observed in the normal blood cells including T cells and B cells (n = 31) [16]. And secondly, analysis of expression microarray data from 38 distinct haematopoietic mature and immature cell populations, confirmed that all B- and T- lineage cell populations tested do express *TES* RNA [49].

Re-expression of *TES* in ALL cell lines by expression plasmid transfection, resulted in fewer surviving cells compared to control experiments. These results are similar to those previously published for *TES* re-expression in other tumour cell lines [20, 25, 22], supporting the proposal that *TES* is a tumour suppressor gene.

We demonstrated that the reduction in *TES*-transfected cell number was in part due to the rapid induction of cell death and in part due to cell cycle arrest. Induction of apoptosis after *TES* re-expression has been reported in other cancer cell lines [22, 25, 20]. For example, Sarti *et al*. reported that *TES* expression induced caspase-dependent or independent apoptosis in breast and uterine cancer cell lines [20]. Gu *et al*. performed similar *TES* re-expression experiments in endometrial carcinoma cell lines and xenografts and reported pro-apoptotic and anti-proliferative effects of *TES* re-expression [22]. Intriguingly, TESTIN expression blocked G1/S progression in the endometrial carcinoma cells compared to an apparent block in G2/M in our B ALL cells, indicating some tissue-specificity for the anti-proliferative effect. In addition, using 7-AAD staining and FACs analysis we document a large number of cells with genome content greater than 4N in our EGFP-*TES*-expressing NALM6 cells. Using confocal microscopy we were able to confirm the presence of large, bi-nucleated, GFP-positive cells suggesting that the pEGFP-TES-transfected cells were able to complete mitosis, but were unable to undergo cytokinesis (S5 Fig).

The mechanism by which TESTIN protein is able to induce rapid cell death and decrease proliferation is unknown, but would likely involve interactions with other proteins via its LIM-binding domains. One likely candidate is the TP53 protein and its pathway, because of its ability to prevent cell division and to induce apoptosis. Furthermore, two recent reports implicate the TP53 pathway as a potential mediator of TESTIN activity. Firstly, Zyxin, a known binding partner of TESTIN, has been reported to modulate the HIPK2/TP53 pathway [37] and secondly, TESTIN was identified as a binding partner for ELL2 [50], and ELL2 is known to bind and inhibit the transactivational activity of TP53 [41]. Accordingly, we hypothesised that the observed anti-tumour activities of TESTIN re-expression in ALL cells may be due to interactions with TP53 protein and function.

However, TESTIN expression did not induce *TP53* RNA expression or increase TP53 protein levels indicating that cell death likely proceeds via a TP53-independent mechanism. Similarly, TP53 is able to influence cell cycle arrest in part via its ability to induce CDKN1A; however, despite observing a sustained increase in *CDKN1A* RNA transcript levels, we observed no increase in CDKN1A protein after pEGFP-TES transfection (Fig 8). Thus, the mechanism by which TESTIN mediates its anti-proliferative and pro-killing activities in B ALL is still to be determined.

## Conclusions

In conclusion, we demonstrate that *TES* gene silencing in ALL and other tumours is abnormal, as fetal and adult tissues are unmethylated at the *TES* promoter. We report that *TES* re-expression in ALL cell lines results in rapid cell death and decreased proliferation of surviving cells, as previously reported for other tumour cells. However, unlike other reports we show that TESTIN re-expression leads to an increase in cells with DNA content greater than 4N and failure to undergo cytokinesis with subsequent polyploidy.

We propose that the observed and desirable anti-tumour effects of TESTIN expression in B ALL and other tumour cells highlight the need to investigate its protein partners and their associated pathways and that this may reveal potential, novel therapeutic targets.

## Supporting Information

S1 Fig *TES* promoter demethylation and decreased Jurkat and MOLT4 cell viability after decitabine exposure. Jurkat and MOLT4 cells were exposed to decitabine (0, 0.5 and 5.0 μM) for 24, 48, 72 and 96 hours. (A) *TES* qualitative RT-PCR: total RNA was mock (-) or reverse transcribed (+) before *TES*-specific amplification with exon-specific primers (exons 5 and 6), (i) Jurkat and (ii) MOLT4. Expected PCR product sizes for cDNA and genomic DNA were 213 bp and 555 bp, respectively (low level contamination can be seen in the mock RT samples from the Jurkat cells). *TES* expression levels, as measured by quantitative RT-PCR assay and calculated relative to PBL cDNA, are recorded under each lane. (B) Viable cell number was measured by WST-1 assay; (i) Jurkat and (ii) MOLT4 (error bars are standard deviations).
(TIF)

S2 Fig Induction of NALM6 cell death after pEGFP-TES or pcDNA-TES transfection. NALM6 cells labelled with Annexin V and VLD were analysed by flow cytometry. Increased numbers of Annexin V and VLD positive NALM6 cells were present 24 hours after transfection with either *TES*-expression plasmid (n = 3; error bars are standard errors).
(TIF)

S3 Fig *TES* transfection reduces proliferation of Jurkat and MOLT4 cell lines. Cells were transfected with mock, pEGFP-C1 or pEGFP-TES expression plasmids, cultured and counted after trypan blue staining; (A) Jurkat (n = 4) and (B) MOLT4 (n = 4)(error bars are standard errors).
(TIF)

S4 Fig Comparison of transfections of NALM6 and Raji cell lines. (A) Comparison of GFP-positive cell numbers after pEGFP-C1 control and pEGFP-TES plasmid transfections of NALM6 and Raji cell lines. Average transfection results showing percent GFP-positive cells present 24 hours post-transfection (NALM6, n = 5; Raji, n = 3). Fewer GFP-positive cells are observed after pEGFP-TES compared to pEGFP-C1 control transfection of NALM6 cells (p<0.01). In contrast, Raji cells did not show this dramatic decrease in percent GFP-positive cells after pEGFP-TES transfection. (B) Using UV microscopy, fewer GFP-positive NALM6 cells were visible after pEGFP-TES transfection than after control pEGFP-C1 transfection at 48 hours post-transfection (*upper panel*). Flow cytometry analysis confirmed that fewer GFP-positive cells were present after pEGFP-TES transfection (16.8% of total) than were present after control transfection (48.2% of total)(*lower panel*).
(TIF)

S5 Fig Composite confocal image of pEGFP-TES transfected and untransfected NALM6 cells. pEGFP-TES transfected NALM6 cells (48 hours post-transfection) were fixed with 2% PFA and labelled with mouse anti-α tubulin and goat anti-mouse AlexaFluor secondary antibody (*red*), before staining with Hoechst (*blue*). The image shown is of a pEGFP-TES transfected cell (large GFP-positive cell) that has two discrete and separate nuclei (confirmed with a set of z-series images, data not shown).
(TIF)

S1 File Sequenom MassARRAY methylation data. Sequenom MassARRAY methylation data for the 48 CpG sites of the *TES* promoter from normal, somatic tissues and lymphoblastoid cell lines.
(XLSX)

## Abbreviations

7-AAD: 7-aminoactinomycin D
ALL: acute lymphoblastic leukaemia
ATCC: American Type Culture Collection
GFP: green fluorescent protein
MFI: median fluorescent intensity
PBL: peripheral blood leukocytes
VLD: Violet Live Dead
VPD: Violet Proliferation Dye
