# Supplementary figures and images for: TESTIN Induces Rapid Death and Suppresses Proliferation in Childhood B Acute Lymphoblastic Leukaemia Cells
Source: PLoS One. 2016 Mar 17;11(3):e0151341. doi: 10.1371/journal.pone.0151341 (PMC4795691; doi:10.1371/journal.pone.0151341)

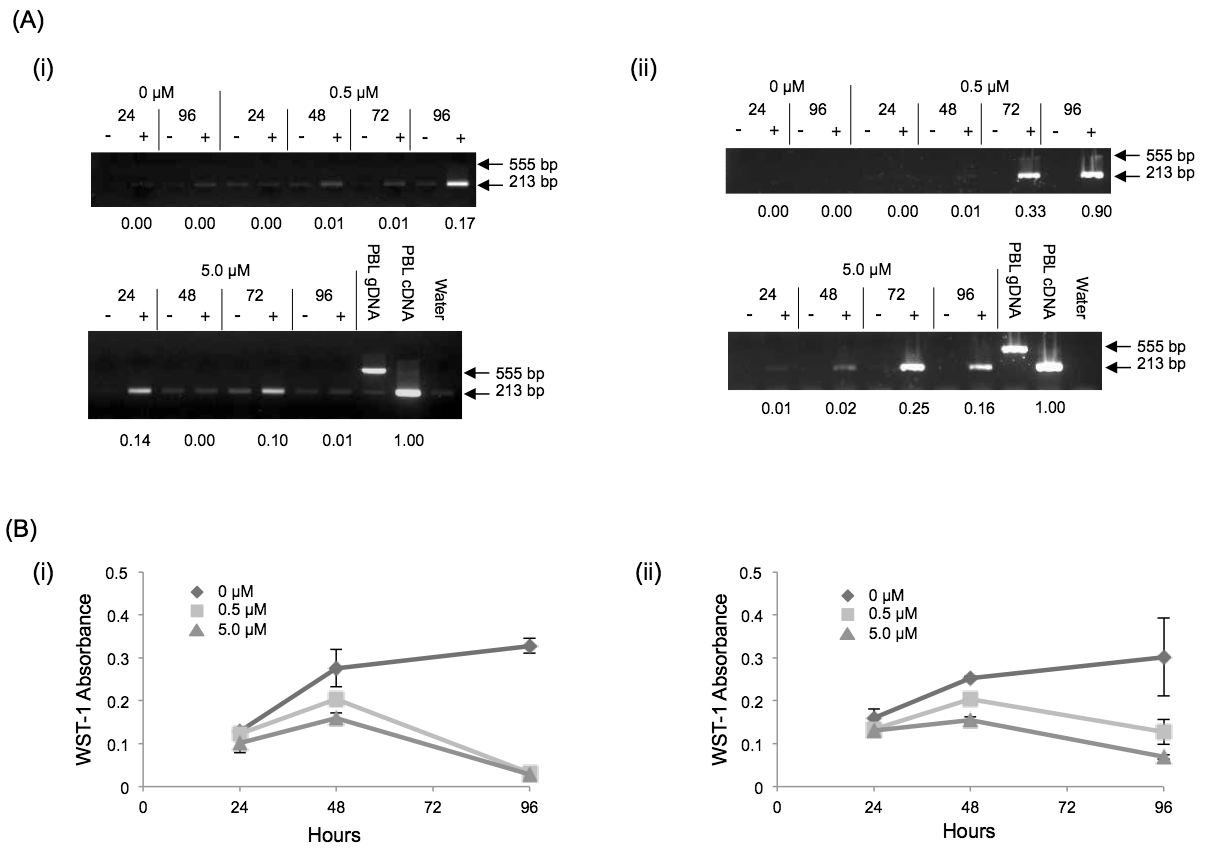

Supplement: S1 Fig — Jurkat and MOLT4 cells were exposed to decitabine (0, 0.5 and 5.0 μM) for 24, 48, 72 and 96 hours. (A) TES qualitative RT-PCR: total RNA was mock (-) or reverse transcribed (+) before TES-specific amplification with exon-specific primers (exons 5 and 6), (i) Jurkat and (ii) MOLT4. Expected PCR product sizes for cDNA and genomic DNA were 213 bp and 555 bp, respectively (low level contamination can be seen in the mock RT samples from the Jurkat cells). TES expression levels, as measured by quantitative RT-PCR assay and calculated relative to PBL cDNA, are recorded under each lane. (B) Viable cell number was measured by WST-1 assay; (i) Jurkat and (ii) MOLT4 (error bars are standard deviations). (TIF) [file pone.0151341.s001.tif]

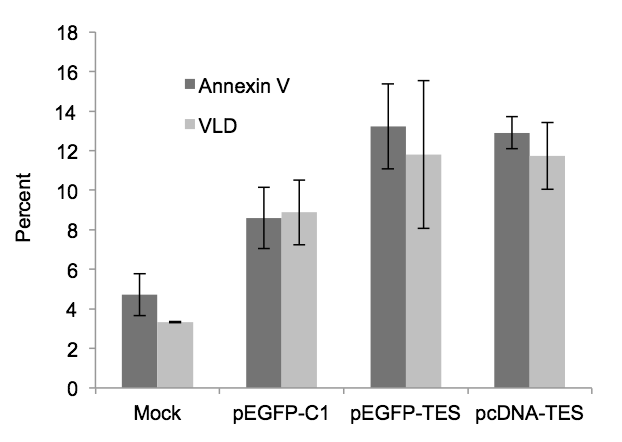

Supplement: S2 Fig — NALM6 cells labelled with Annexin V and VLD were analysed by flow cytometry. Increased numbers of Annexin V and VLD positive NALM6 cells were present 24 hours after transfection with either TES-expression plasmid (n = 3; error bars are standard errors). (TIF) [file pone.0151341.s002.tif]

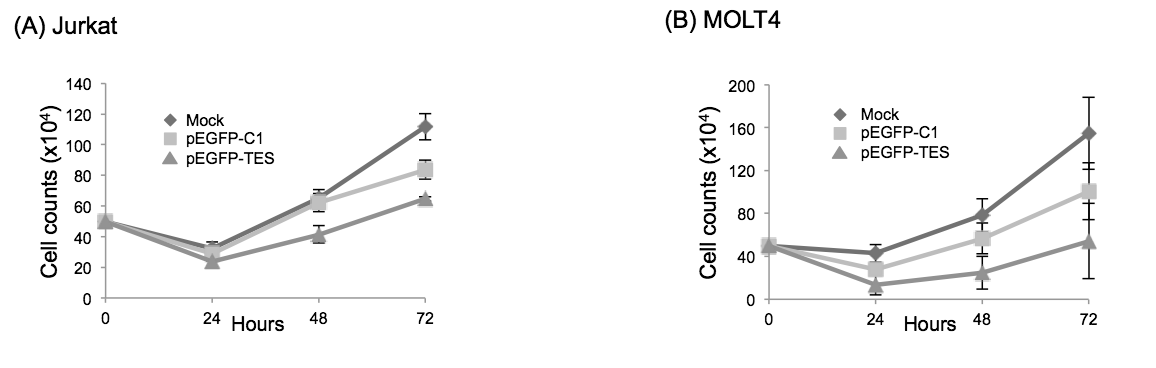

Supplement: S3 Fig — Cells were transfected with mock, pEGFP-C1 or pEGFP-TES expression plasmids, cultured and counted after trypan blue staining; (A) Jurkat (n = 4) and (B) MOLT4 (n = 4)(error bars are standard errors). (TIF) [file pone.0151341.s003.tif]

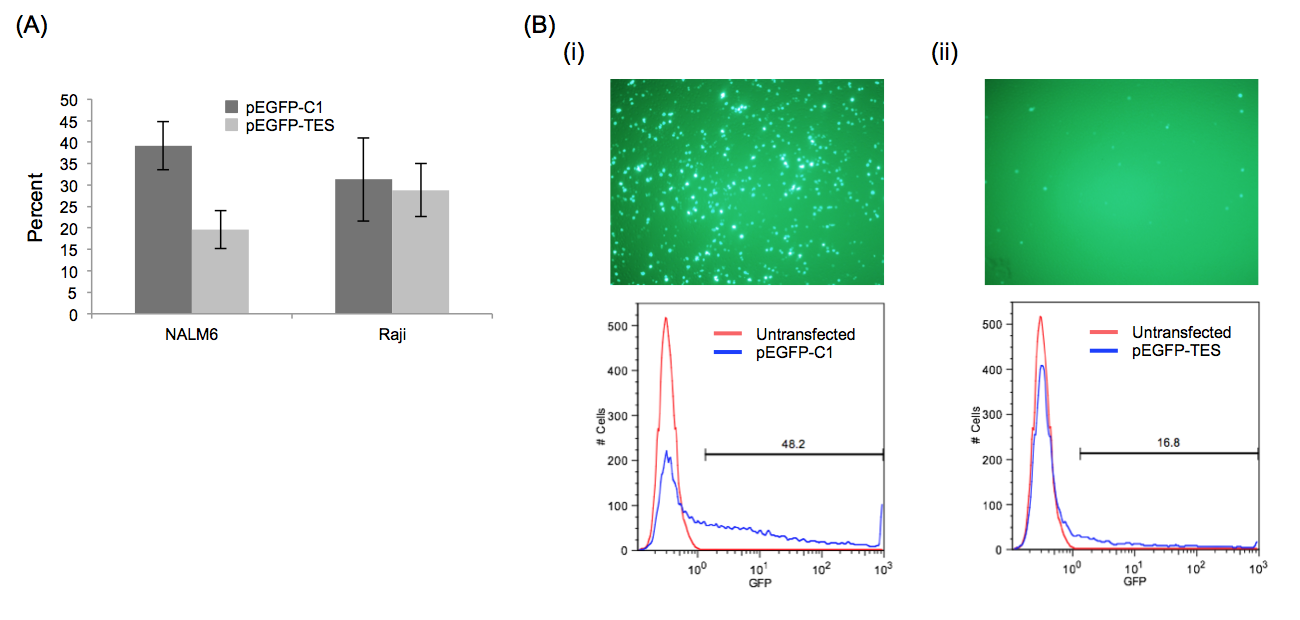

Supplement: S4 Fig — (A) Comparison of GFP-positive cell numbers after pEGFP-C1 control and pEGFP-TES plasmid transfections of NALM6 and Raji cell lines. Average transfection results showing percent GFP-positive cells present 24 hours post-transfection (NALM6, n = 5; Raji, n = 3). Fewer GFP-positive cells are observed after pEGFP-TES compared to pEGFP-C1 control transfection of NALM6 cells (p<0.01). In contrast, Raji cells did not show this dramatic decrease in percent GFP-positive cells after pEGFP-TES transfection. (B) Using UV microscopy, fewer GFP-positive NALM6 cells were visible after pEGFP-TES transfection than after control pEGFP-C1 transfection at 48 hours post-transfection (upper panel). Flow cytometry analysis confirmed that fewer GFP-positive cells were present after pEGFP-TES transfection (16.8% of total) than were present after control transfection (48.2% of total)(lower panel). (TIF) [file pone.0151341.s004.tif]

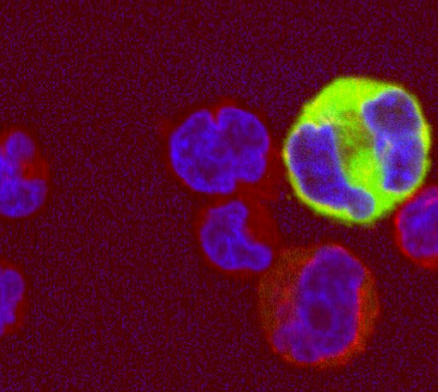

Supplement: S5 Fig — pEGFP-TES transfected NALM6 cells (48 hours post-transfection) were fixed with 2% PFA and labelled with mouse anti-α tubulin and goat anti-mouse AlexaFluor secondary antibody (red), before staining with Hoechst (blue). The image shown is of a pEGFP-TES transfected cell (large GFP-positive cell) that has two discrete and separate nuclei (confirmed with a set of z-series images, data not shown). (TIF) [file pone.0151341.s005.tif]
